# Vibration and Noise Analysis and Experimental Study of Rail Conveyor

**DOI:** 10.3390/s23104867

**Published:** 2023-05-18

**Authors:** Nini Hao, Xinming Sun, Mengchao Zhang, Yuan Zhang, Xingyu Wang, Xiaoting Yi

**Affiliations:** 1 College of Mechanical and Electronic Engineering, Shan Dong University of Science and Technology, Qingdao 266590, China; 2 Libo Heavy Industries Science and Technology Co., Ltd., Tai’an 271000, China

**Keywords:** rail conveyor, vibration noise, spectrum analysis, field experiments

## Abstract

The rail conveyor is a new type of energy-saving system for the long-distance transportation of bulk materials. Operating noise is an urgent problem that the current model faces. It will cause noise pollution and affect the health of workers. In this paper, the factors causing vibration and noise are analyzed by modeling the wheel-rail system and the supporting truss structure. Based on the built test platform, the system vibration of the vertical steering wheel, the track support truss, and the track connection were measured, and the vibration characteristics at different positions were analyzed. Based on the established noise and vibration model, the distribution and occurrence rules of system noise under different operating speeds and fastener stiffness conditions were obtained. The experimental results show that the vibration amplitude of the frame near the head of the conveyor is the largest. The amplitude under the condition of 2 m/s running speed at the same position is 4 times that under the condition of 1 m/s. At different welds of the track, the width and depth of the rail gap have a great influence on the vibration impact, which is mainly due to the impact of the uneven impedance at the track gap, and the greater the running speed, the more obvious the vibration impact. The simulation results show the trend of noise generation, the speed of the trolley, and the stiffness of the track fasteners have a positive effect on the generation of noise in the low-frequency region. The research results of this paper will play an important role in the noise and vibration analysis of rail conveyors and help to optimize the structure design of the track transmission system.

## 1. Introduction

Belt conveyors play an important role in solid bulk material transport systems and have the advantages of long-distance continuous transportation, large transportation volume, and small environmental impact. Research on belt conveyors is increasingly inclined to intelligent-monitoring and energy-saving technologies [1,2,3,4,5,6,7,8]. However, traditional belt conveyors are associated with energy consumption due to large running resistance. The rail conveyor recently developed [5,6,7] effectively combines the benefits of low friction resistance, low energy consumption for wheel-track transportation, continuous material transport, and large capacity for belt conveyors. It is a new type of energy-saving conveyor system with great development potential.

In the rail conveyor system, the conveyor belt rests on the support carriages, there is no relative movement between the carriages and the belt, and the support carriages run on the track by rolling the wheels. Due to the elimination of the indentation rolling resistance and belt and bulk material flexure resistance within the conveyor system, an approximate reduction of 50% in energy consumption can be achieved. Theoretical and experimental results have shown that under the condition of the same conveyor power, the transportation distance of the rail conveyor can reach 1.5–2 times that of the conventional belt conveyors, and the longer the transportation distance, the more obvious the energy-saving effect [8,9].

The introduction of the wheel-rail system is a technological breakthrough and improvement in the design of belt conveyors, which effectively overcomes the limitations of traditional belt conveyors and can achieve very low resistance operation. However, there is a significant dynamic interaction between wheels and tracks in the operation of rail conveyors. Therefore, effectively reducing the vibration and noise of the rail conveyor system is very necessary for the optimal design of the system structures [10].

At present, the research and development of the rail conveyor system is still in its infancy, and there is no complete research literature on the vibration and noise of the rail conveyor. There are many literature reports on the research of wheel-track vibration and noise, mainly related to the wheel-track vibration and noise of railway transportation. Research topics include but are not limited to wheel-rail noise, aerodynamic noise, vibration and noise control, wheel-rail interaction, numerical simulation, and experiment, measurement of vibration and noise, etc. Remington [11] gave a comprehensive explanation of the mechanism of wheel-track rolling noise and established a frequency-domain model for predicting wheel-track rolling noise. Based on Remington’s work, Thompson [12] generalized and further improved the rolling noise prediction model. The mechanism of rolling noise is due to the exciting force generated by the rough surfaces of the track and wheel during the movement, which causes vibration and noise between the wheels and the rails [13,14,15]. Li [16,17] established a frequency-domain theoretical model of a vehicle-track coupling system considering the multiple-wheel effect to study the influence of different track structures on the vibration and noise of elevated concrete box girders. Mandula [18] proposed an improved calculation model for the prediction of levels of noise from trams. Gu [19] explored the noise radiation spectrum characteristics of composite bridges by building vibration and noise prediction models for railway composite bridges. Liang [20] studied the vibration and noise reduction mechanism and characteristics of three typical vibration-damping tracks on long-span steel bridges. Liu [21] predicted vibration and structure-borne noise in the frequency domain by building a finite element-boundary element method (FE-BEM) model of a track bridge and performed field vibration and noise tests. Hao [22] proposed a Hurst exponent-based adaptive line enhancer (ALE) acoustic emission detection method for weak rail crack signals in a high-speed railway strong wheel-rail rolling noise environment. He [23] conducted parametric research on key design parameters such as rubber material parameters, number of rubber blocks, and gap rubber ratio by establishing a wheel-rail noise prediction model. Hou [24] realized the joint simulation and prediction of wheel-rail system vibration, rolling noise, and aerodynamic noise by establishing a high-speed railway wheel-rail rolling noise model. Torstensson [25] used a combination of time-domain simulation of nonlinear vertical dynamic vehicle-track interaction and a hybrid prediction model using linear frequency domain to calculate and predict sound pressure levels for railway crossing wheel-rail impact loads and noise. Mazilu [26] studied the influence of rail pad elastic properties on wheel-rail vibration from the perspective of wheel-rail contact force and wheel-rail acceleration by establishing a non-uniform foundation model. Song [27] developed a numerical model of a U-shaped girder bridge with piers and frequency-dependent stiffness fasteners (FDSF) and discussed the acoustic contribution and sound pressure distribution of the piers and stiffness fasteners. Chiba [28] proposed a numerical simulation method for the wheel-rail system contact problem using fuzzy logic and predicted the resonance noise in the wheel-rail contact process of rail transit. Yang [29] studied the wheel-rail collision problem of insulated track joints used in embedded track systems both numerically and experimentally.

Since the rail conveyor is a new type of conveyor system, related research work has been less reported. In this work, based on the above-reviewed theory and experiment studies for locomotive wheel-track coupling vibration and noise, some factors affecting the vibration and noise of the rail conveyor system are discussed first, and related mechanics models and equations are provided. The on-site vibration and noise testing methods for a prototype rail conveyor system are then discussed, the test platform is set up, and the measurements for the vibration and noise characteristics at several locations of the prototype rail conveyor are carried out. The obtained experimental data provide a valuable reference for improving the vibration and noise performance of the rail conveyor system.

## 2. Factors Affecting System Vibration and Noise

### 2.1. Influence of Wheel-Track System on Vibration and Noise

The noise of the rail conveyor is mainly affected by the wheel-track contact vibration and the vibration of the support carriages running on tracks, which may be caused by the following reasons:

- The influence of track surface irregularity: Long-term operation of the rail conveyor may cause the track surface to wear, rough and uneven, or to have dark pits, which makes the track and the carriage wheels not smoothly contact, resulting in vibration between wheels and tracks;
- The effect of speed on vibration: Vibration is caused by the interaction between wheels and tracks when the support carriages pass through the tracks at a certain speed;
- Influence of rail weld joints: Long-running of the rail, due to the impact of the wheels, will lead to cracking of the weld at the rail joint, and the weld will appear low joint. The low joint phenomenon is common in the seamed structure, and the specific performance is that the joint at the weld seam sinks.

In view of the above-mentioned factors influencing system vibration and noise, the corresponding analysis models introduced according to relevant literature are summarized as follows:

1. Track surface irregularity model

**Figure 1:**
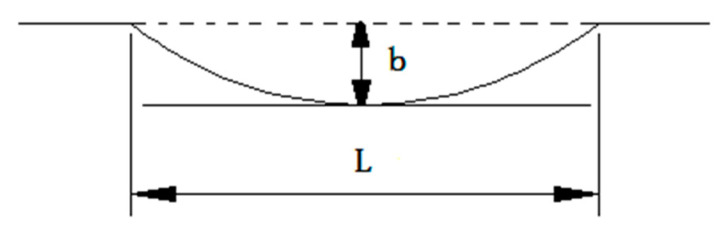
Track irregularity model.

Assuming that the uneven track surface can be described by the cosine function shown in Figure 1, the resulting harmonic excitation can be expressed as:

(1) $$Z_{0}(t)=\frac{1}{2}b(1-\cos\omega t)\left(0\leq t\leq L/V\right)$$

where ω=2πV/L is the irregularity wavelength and the irregular wave depth.

2. Speed model

Since the conveyors do not need to operate at high speed, the support carriages usually run at low speed, and the low-speed impact model can be expressed as:

(2) $$V_{0}=(1+\gamma )\frac{L}{2R}V$$

where L is the scar length of the support carriage wheel, γ is the coefficient of wheel rotation inertia converted to reciprocating inertia, and R is the wheel radius.

It can be seen from the above model that the impact speed of the support carriage during operation is proportional to the length of the flat scar on the wheel and the speed of the support carriage.

3. Track weld joint model

**Figure 2:**
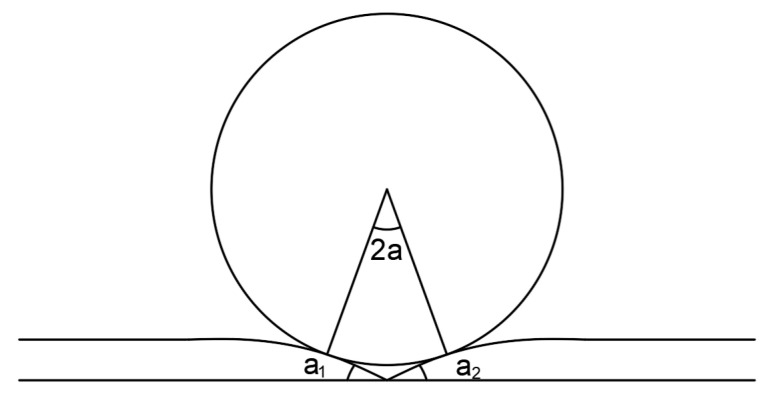
Weld joint model.

Weld low joints often appear in the belt seam structure shown in Figure 2, and the impact velocity at the low joint is expressed by the product of the angle $a_{1}$, $a_{2}$ at the joint and the speed *V* of the supporting vehicle:

(3) $$V_{0}=2aV=\left(a_{1}+a_{2}\right)V$$

where 2a is the track weld low joint total angle.

### 2.2. The Influence of Truss Structure on Track Noise

1. Truss structure model

The wheel-track interaction is the main source of vibration. For the rail conveyor system, the support carriages are placed on the tracks, and the tracks are fixed on the truss. When support carriages are running, the vibrations of the wheel-rail system and the support carriages will also be transmitted to the truss structure, resulting in coupled vibration of the truss. The vibration problem of the truss will become even more prominent as its length, height, and span increase. In addition, with the long-term use of conveyors, the aging and corrosion of the steel structure will also cause a change in the dynamic characteristics of the truss structure, making the vibration-related problems more obvious with the increase in time. It can be seen that the truss of the rail conveyor is used as the main body of the load-bearing, and its own vibration characteristics have a great influence on the operation, vibration, and noise characteristics of the conveyor system.

**Figure 3:**
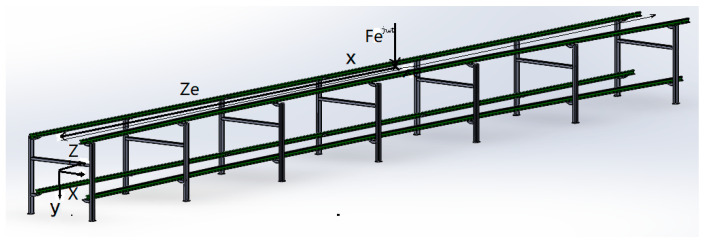
Truss structure model.

The model of the conveyor truss structure can be shown in Figure 3. Due to the angles of the inner and outer tracks in the turning section of the tracks, the centerline of the track deviates from its symmetrical center. When the support carriages pass through the turning section of the tracks, different axles of the wheels of the support carriages will appear, causing torsional and bending vibrations of the tracks at the same time. Therefore, in the truss mechanics model, torsion and bending deformation need to be considered.

According to the above analysis, we have carried out a theoretical study on the vibration problem of the system when the inner and outer orbits are angled. Assuming that a simple harmonic load $Fe^{i\omega t}$ is applied at the position of $z=z_{e}$ on a rail, F is the load amplitude, ω is the excitation circle frequency, and i is a unit imaginary number, the differential equations for the bending and torsional vibration of the truss are:

(4) $$\rho A\frac{\partial^{2}y}{\partial t^{2}}+EI(1+i\eta )\frac{\partial^{4}y}{\partial t^{4}}=Fe^{i\omega t}\delta (z-z_{e})$$

(5) $$\rho I_{p}\frac{\partial^{2}\theta }{\partial t^{2}}-GJ_{t}(1+i\eta )\frac{\partial^{2}\theta }{\partial z^{2}}=FX_{e}e^{i\omega t}\delta (z-z_{e})$$

where y is the vertical displacement of the truss, θ is the truss torsional angle, ρ is material density, A is the truss cross-sectional area, I is (vertical bending) inertia moment of the truss area, E is young’s modulus of the truss material, G is the shear modulus of the truss materials, η is the loss factor, $I_{p}$ is the polar moment of inertia of section, and $J_{t}$ is the truss torsional moment of inertia.

2. Track-truss coupling system model

**Figure 4:**
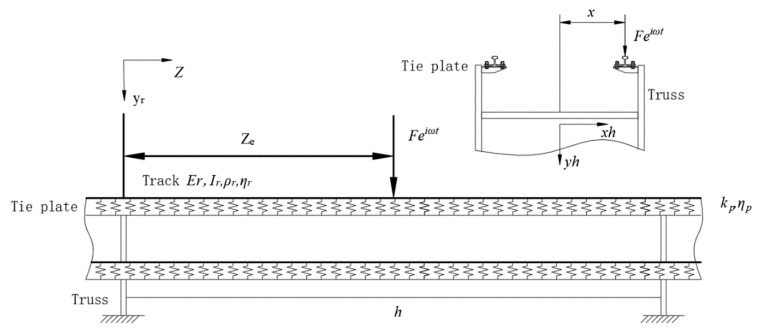
Track-truss coupling system model.

The track is fixed on the truss by fasteners. To simplify the model, the track is regarded as an infinite Euler-Bernoulli beam. It can be seen from the schematic diagram of the track shown in Figure 4 that the tracks of the conveyor have upper and lower levels. In this work, only the upper track is studied to simplify the calculation, ignoring the mutual influence between the upper and lower tracks, and the fasteners are treated as a spring with a damping structure.

In this paper, the dynamic flexibility method is used to study the vertical vibration of the track. The dynamic flexibility, r is defined as the displacement caused by the unit harmonic load,

(6) $$r=\frac{Y(z)}{F(\omega )}$$

where F(ω) is the harmonic load and Y(z) is the displacement induced.

For linear vibration, the system satisfies the principle of superposition. The displacement of the track is equal to the superposition of the displacement caused by all the forces acting on it, and the displacement caused by each force is the product of this force and the corresponding dynamic flexibility. Therefore, the steady-state displacement amplitude of the track r is:

(7) $$Y_{r}(z)=F\alpha (z,z_{e})-\sum_{n=1}^{N}F_{n}\alpha (z,z_{n})$$

where $\alpha (z,z_{n})$ is the dynamic flexibility function of the track at the position of the *n*-th fastener. The magnitude of the steady-state displacement caused by a unit harmonic load at $z=z_{2}$.

The steady-state displacement response of truss h is:

(8) $$Y_{h}(x_{h},z_{h})=\sum_{m=1}^{Nr}F_{hm}\gamma (x_{h},z_{h};x,z_{hm})$$

where $F_{hm}$ is the force acting on the truss by the track at the position of the *m*-th fastener; $x_{h}$ and $z_{h}$ are the lateral displacement and longitudinal displacement of the truss in the local coordinate system; $z_{hm}$ is the longitudinal position of the *m*-th fastener in the local coordinate system of the truss; x is the lateral position of the track in the local coordinate system; γ is the dynamic flexibility coefficient of the truss.

When $n=(h-1)N_{r}+m$, $F_{n}$ and $F_{hm}$ correspond to the same track fastener, the fastener Displacement is:

(9) $$Y_{kn}=\frac{F_{n}}{k_{p}(1+i\eta_{p})}=\frac{F_{hm}}{k_{p}(1+i\eta_{p})}=Y_{r}(z_{n})-Y_{h}(x,z_{hm})$$

Substituting the Equations (6) and (7) into (8), we obtain:

(10) $$\frac{F_{n}}{k_{p}(1+i\eta_{p})}=\frac{F_{h}m}{k_{p}(1+i\eta_{p})}=F\alpha (z_{n},z_{e})-\sum_{n=1}^{N}F_{k}\alpha (z_{n}-z_{k})-\sum_{l=1}^{N_{p}}F_{h}{}_{l}\gamma (x,z_{h}{}_{m};x,z_{h}{}_{l})$$

where $\eta_{p}$ is the loss factor; $k_{p}$ is the rigidity of track fasteners; $z_{h}{}_{l}$ is the position that causes the steady-state displacement of the truss.

After proper arrangements, Equations (8) and (9) can be written into the following matrix form:

(11) RF=Q

where R is a 2*N* × 2*N* dimensional system dynamic flexibility matrix, which is composed of orbital dynamic flexibility α, fastener dynamic flexibility $1/k_{p}(1+i\eta_{p})$ and truss dynamic flexibility γ; F is the 2*N*-dimensional column vector composed of the force of the fastener on the track, and other elements in the matrix are all zero; Q is the 2*N*-dimensional load column vector, and the value is $F\alpha (z_{pn}-z_{e})$, *n* = 1, 2, …, *N*.

Substituting the fastener’s force obtained by Equation (10) into Equation (7), the velocity admittance can be obtained as:

(12) $${\dot{Y}}_{r1}(z_{e})=i\omega Y_{r1}(z_{e})$$

According to the harmonic response analysis, analyze and calculate the vibration characteristics such as admittance, use ANSYS software to analyze the harmonic response of the structure, and import the structure analyzed by ANSYS into MATLAB software for analysis, so as obtain the admittance of the structure.

3. Wheel-rail radiation noise prediction model

According to the above analysis, the noise prediction model of wheel-rail conveyor was established, and the influence of different parameters of the conveyor on the noise was analyzed. In the frequency spectrum of the wheel rail, we mainly analyze the response represented by 1/3 times the frequency band. The radial vibration velocity power of the wheel and the track at the wheel-rail contact point can be obtained by analyzing the wheel-rail radial vibration power spectrum:

(13) $$S_{{\tilde{Y}}_{W}}^{PC}(\omega )=|H_{W}(\omega ){|}^{2}\omega^{2}|H(k){|}^{2}[B_{W}(k)+B_{R}(k)]$$

(14) $$S_{{\tilde{Y}}_{R}}^{PC}(\omega )=|H_{R}(\omega ){|}^{2}\omega^{2}|H(k){|}^{2}[B_{W}(k)+B_{R}(k)]$$

where $S_{{\tilde{Y}}_{W}}^{PC}(\omega )$ is the radial vibration velocity power spectrum of the supporting vehicle wheel at the wheel-rail contact point; $S_{{\tilde{Y}}_{R}}^{PC}(\omega )$ is the radial vibration velocity power spectrum of the conveyor track at the wheel-rail contact point; ω is the time-frequency; $B_{W}(k)$ is the integral of the estimated value of the spectrum of the wheel at the point of contact; $B_{R}(k)$ is the integral of the spectral estimate of the orbit at the contact point; H(k) is the transfer function that filters out the non-smooth components; k is the space wave number, $k=\frac{\omega }{v}$ (v is the speed of the supporting vehicle); $H_{W}(\omega )$, $H_{R}(\omega )$ is the wheel-rail impedance frequency response function; as follows:

(15) $$H_{W}(\omega )=\frac{Z_{R}}{Z_{W}+Z_{R}-j\omega Z_{W}Z_{R}/K_{C}}$$

(16) $$H_{R}(\omega )=\frac{Z_{W}}{Z_{W}+Z_{R}-j\omega Z_{R}Z_{W}/K_{C}}$$

where $Z_{W}$ is the impedance at the point of radial velocity supporting the wheel of the vehicle; $Z_{R}$ is the impedance at the orbital radial velocity point; $K_{C}$ is the Contact stiffness coefficient between wheel and track.

To predict the vibration and noise of the follow-up conveyor, we introduce an important index of track vibration, the track vibration attenuation coefficient:

(17) $$\eta_{RV}=-\frac{\Delta_{v}}{2k_{RV}}$$

where $k_{RV}$ is the orbital bending vibration wavenumber; $\Delta_{v}$ is the ratio of the distance of the conveyor track vibration attenuation to the noise attenuation.

The sound radiation coefficient of the track and the wheel refers to the ratio of the sound energy radiated from the vibration sound energy of the wheel or track to the medium by the interaction of the structural vibration:

(18) $$\sigma_{WR}=\frac{2}{1+{\left(f_{w}/f\right)}^{2}}$$

(19) $$\sigma_{RV}=\frac{2}{1+{\left(f_{R}/f\right)}^{3}}$$

where $\sigma_{WR}$ is the radial radiation efficiency of the supporting vehicle wheel; $f_{w}$ is the acoustic radiation critical frequency of the supporting vehicle wheel; f is the noise frequency; $\sigma_{RV}$ is the vertical vibration radiation efficiency of the track; $f_{R}$ is the orbital acoustic radiation critical frequency.

From the above formula, it can be deduced that the average power spectrum of the track when the supporting vehicle passes the track in the time *T* is:

(20) $$S_{{\dot{Y}}_{R}}^{AVG}(\omega )=\frac{N}{2\eta_{RV}k_{RV}VT}[1-exp(-\eta_{RV}k_{RV}VT)]S_{{\tilde{Y}}_{R}}^{PC}$$

where N is the number of pairs of wheels of the supporting vehicle; $\eta_{RV}$ is the conveyor track attenuation coefficient; $k_{RV}$ is the orbital vibration bending wavenumber; V is the Conveyor running speed.

When calculating the average noise of the wheel, the ground reflection is ignored, and the wheel is assumed to have no lateral vibration; the number of pairs of supporting vehicles is *N*, and the sound pressure level spectrum of the radial vibration radiation of the supporting vehicle wheel beside the conveyor line is

(21) $$L_{PWR}(\omega )=10lg\left\{\frac{N}{dVT}\frac{{\left(\rho c\right)}^{2}}{P_{0}^{2}}\left[\sigma_{WR}A_{WR}S_{{\dot{Y}}_{W}}^{AVG}(\omega )\right]\right\}$$

where $A_{WR}$ is the radial radiation area of the supporting vehicle wheel; d is the measure of the distance of the point from the track; T is the intercepted passing time of the supporting vehicle; $\sigma_{WR}$ is the wheel radial radiation efficiency; ρ is the air density; c is the speed of sound in the air; $S_{{\dot{Y}}_{W}}^{AVG}$ is the average power spectrum of wheel radial vibration; $P_{0}^{}$ is the reference sound pressure, the value is 2 × 10^5^ Pa.

Assuming that there are *N* pairs of supporting vehicle wheels passing the track, the sound pressure level spectrum of the average noise of the vertical vibration of the conveyor track is

(22) $$L_{PVR}(\omega )=10lg\left\{\frac{N}{d}\frac{{\left(\rho c\right)}^{2}}{P_{0}^{2}}\sigma_{RV}r_{F}S_{{\dot{Y}}_{R}}^{AVG}(\omega )\right\}$$

where $\sigma_{RV}$ is the vertical vibration radiation efficiency of the conveyor track; $r_{F}$ is the track width; $S_{{\dot{Y}}_{R}}^{AVG}$ is the vertical vibration power spectrum of the track.

The average noise radiated by the wheel rail of the conveyor next to the line can be regarded as the superposition of the average noise of the wheel and the track, and the wheel-rail noise of the conveyor at the observation point is

(23) $$L_{ptotal}(\omega )=10lg\left\{\frac{N}{2d}\frac{{\left(\rho c\right)}^{2}}{P_{0}^{2}}\sigma_{RV}r_{F}S_{{\dot{Y}}_{R}}^{AVG}(\omega )+\frac{N}{2dVT}\frac{{\left(\rho c\right)}^{2}}{P_{0}^{2}}\sigma_{WR}A_{WR}S_{{\dot{Y}}_{W}}^{AVG}(\omega )\right\}$$

## 3. Methods and Procedures for Vibration and Noise Measurements

**Figure 5:**
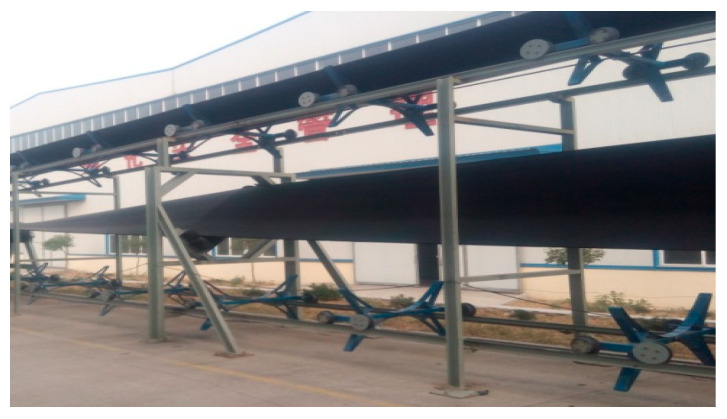
Conveying section of prototype rail conveyor.

We built a prototype rail conveyor, which consists of head, tail, and middle sections. The head and tail sections of the system are the vertical turnaround wheels, and the middle section is the turning part. The conveying section of the prototype system is shown in Figure 5. The test is carried out in the following steps:

1. Establish a test system and determine the test scheme

The test objects are trusses, tracks, etc., and the installation position of the sensor on the truss is determined according to the specific requirements of each test. Vibration and noise sensors are used to measure the vibration response and noise value of the conveyor at different locations and under different conditions. The collected vibration signal is analyzed in the frequency domain by the Fourier transform, and the vibration signal is introduced into the established wheel-rail coupling model. The model is used to simulate and analyze the running noise of the wheel-rail belt conveyor. The obtained results are analyzed and fitted with the collected noise signals to obtain the generation trend of the vibration noise of the wheel-rail conveyor and the impact of the noise under different conditions.

2. Instrument configuration process

**Figure 6:**
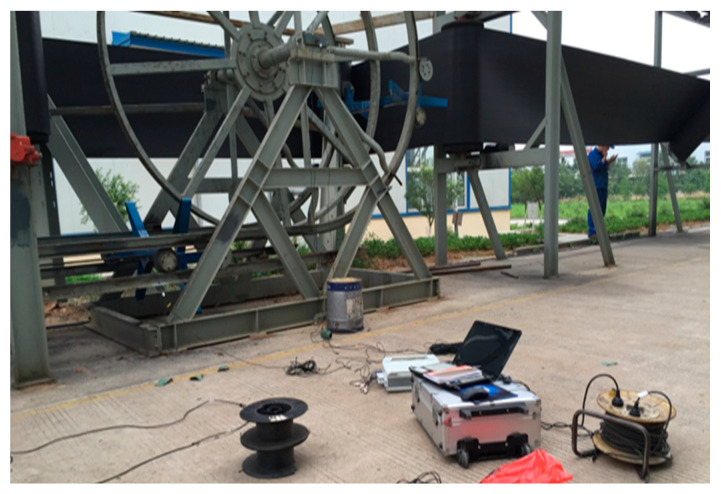
Test site.

For measurement, the Donghua DH5922 16-channel signal acquisition instrument produced by China Jiangsu Donghua Testing Technology Co., Ltd. with the sampling frequency *Fs* = 10 kHz is selected, and the vibration sensors used include DH131E, DH311E, DH186, AC133-1D and the noise sensor GRAS46AE. Figure 6 shows the test site for the measurement. The system acquisition software is DHDAS large-scale dynamic signal acquisition and analysis software, which is used to detect and record waveform data, signal processing, calculation analysis, and the output of data and graphics.

After completing the connection of the instrument, according to the Nyquist sampling law, the sampling frequency should be at least twice the frequency of the signal under test, so that the digital signal after sampling completely retains the information in the original signal without aliasing. The sampling frequency *Fs* = 10 is selected.

3. Collect experimental data

Run the conveyor and obtain actual measurement data. Before taking a measurement, connect the signal line plug of the adapter to the instrument reliably, start the detection instrument, detect the connection of the equipment line, calibrate the measurement of the equipment, and check the transmission error of the equipment. The measurement time is set to a single sampling time of 600 s, the vibration acceleration sensor is installed on the surface of the structure, and the noise sensor is tested at the distance of 1.5 m from the transportation line.

## 4. Test Results and Analysis

### 4.1. Vibration Characteristics Analysis of Conveyor at Different Positions

To comprehensively test the vibration characteristics of the conveyor, the test is divided into three parts. The measured positions or working conditions were the axial and vertical directions of the track and the truss and the locations of the different welds of the track. In view of the above three situations, the sensor layout and field test were carried out at the different measuring points, detailed as follows.

1. Locations above the vertical turnaround wheel and the track truss of the machine head

**Figure 7:**
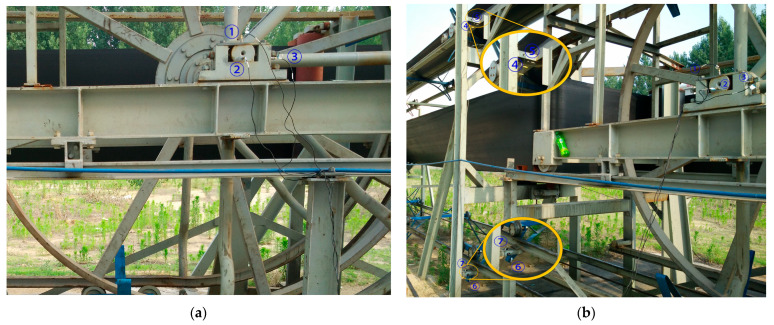
The layout of measurement points on the vertical turnaround wheel beam and track truss: (**a**) ①, ②, ③ measuring point layout; (**b**) ④, ⑤, ⑥, ⑦ measuring point layout.

**Figure 8:**
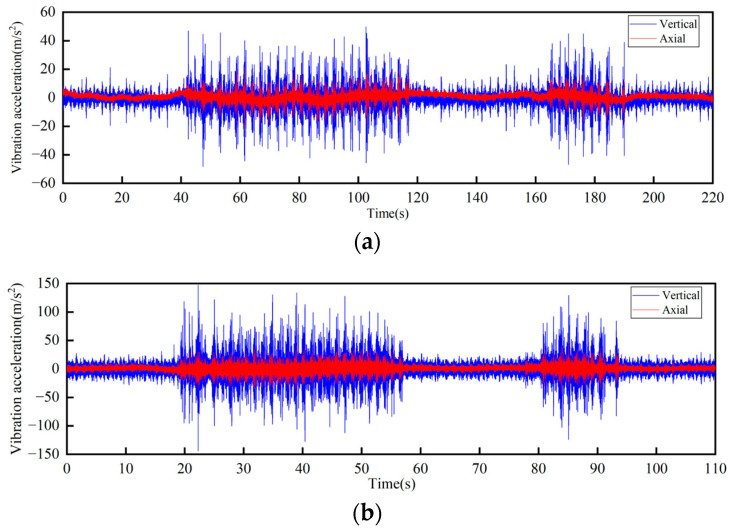
Time-domain curves of vibration acceleration measured at the rotating shaft above the side beam of the vertical turnaround wheel of the machine head: (**a**) Speed 1 m/s; (**b**) Speed 2 m/s.

**Figure 9:**
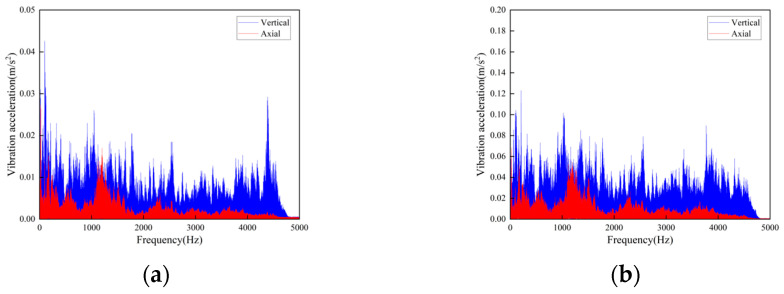
Frequency domain curves of vibration acceleration measured at the rotating shaft above the side beam of the vertical turnaround wheel of the machine head: (**a**) Speed 1 m/s; (**b**) Speed 2 m/s.

**Figure 10:**
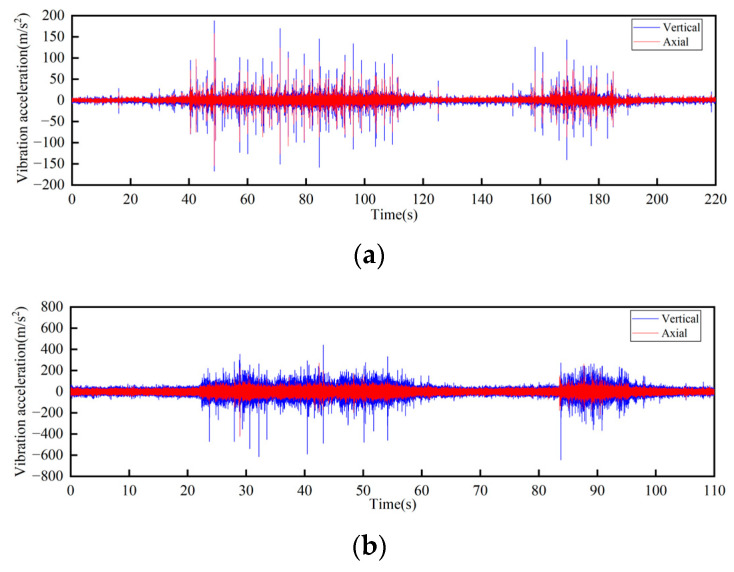
Time-domain curves of vibration acceleration measured at the connection of the upper track truss: (**a**) Speed 1 m/s; (**b**) Speed 2 m/s.

**Figure 11:**
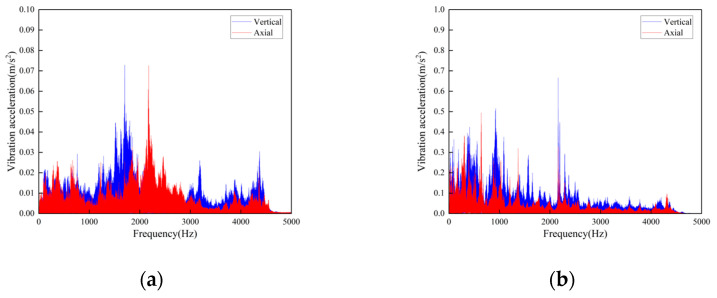
Frequency domain curves of vibration acceleration measured at the connection of the upper track truss: (**a**) Speed 1 m/s; (**b**) Speed 2 m/s.

**Figure 12:**
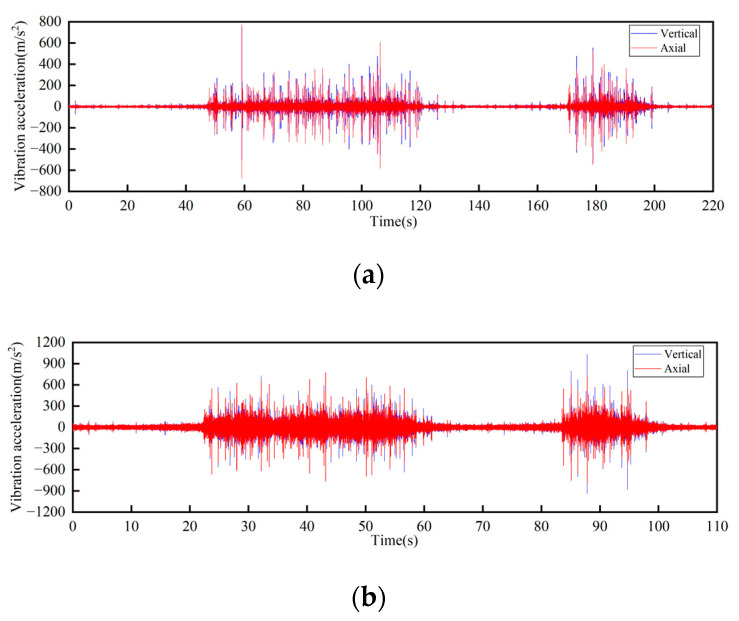
Time-domain curves of vibration acceleration measured at the connection of the lower track truss: (**a**) Speed 1 m/s; (**b**) Speed 2 m/s.

**Figure 13:**
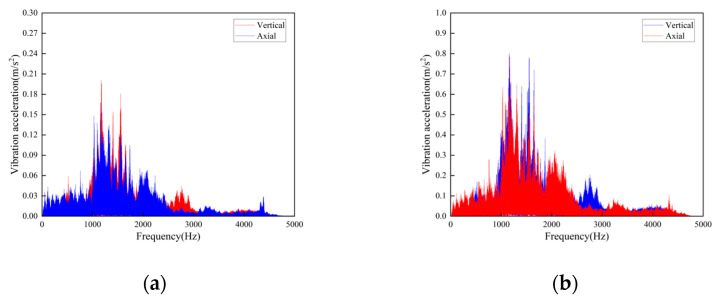
Frequency domain curves of vibration acceleration measured at the connection of the lower track truss: (**a**) Speed 1 m/s; (**b**) Speed 2 m/s.

In the test, a total of seven sensors were arranged on the track and the truss. Three of the sensors DH131E were arranged above the lateral beam of the vertical turnaround wheel, as shown in Figure 7a at the locations ①, ②, and ③ to measure the vibration characteristics of the track axial and the vertical structures, respectively; near the head vertical turnaround wheel truss, two DH131E vibration sensors were arranged at the horizontal and vertical directions of the leg below the upper track, respectively, as seen in Figure 7b at ④ and ⑤; and the other two sensors DH311E were arranged at the connection between the lower track and the truss, as seen in Figure 7b ⑥ and ⑦. The signal output of the rail conveyor at the speeds of 1 m/s and 2 m/s, respectively, were collected. The installation location information of each measuring point sensor is listed in Table 1. By performing Fourier transform on the measurement results, the vibration signals are analyzed in the frequency domain and time domain, respectively. The measurement results are shown in Figure 8, Figure 9, Figure 10, Figure 11, Figure 12 and Figure 13.

According to the test results, the vibration characteristics at different measuring points can be discussed as follows:

- In the part of the machine head, the vibration of the truss is obviously stronger than that of the vertical turnaround wheel under the same speed, and the vibration of the truss is mainly distributed in the frequency range of 1000–2000 Hz.
- The vertical vibration at the shaft of the side beam of the vertical turnaround wheel is greater than the axial vibration. At the track truss connections, the vibration amplitude under the speed of 2 m/s is about 4 times that at 1 m/s, and the vibration amplitude of the lower track truss connection is about twice that of the upper one.
- Unlike the vibration property on the track, the vertical and horizontal vibration intensity at the connection between the track and truss is at the same level.

2. Track connections

**Figure 14:**
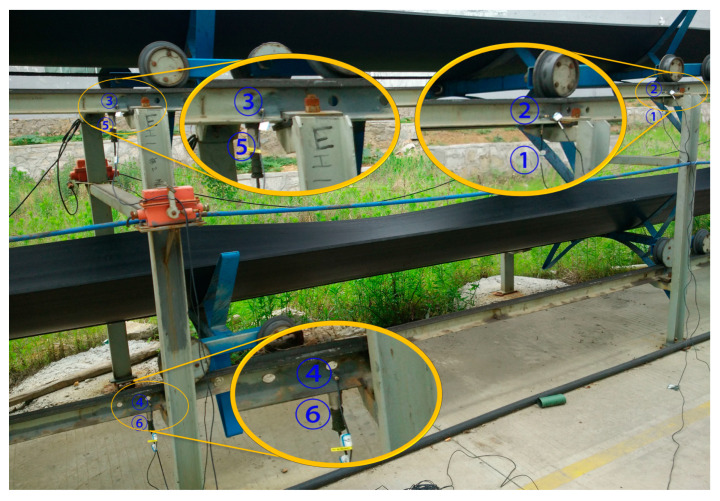
The layout of measuring points at rail joints: ①, ②, ③, ④, ⑤ and ⑥ measuring point layout.

**Figure 15:**
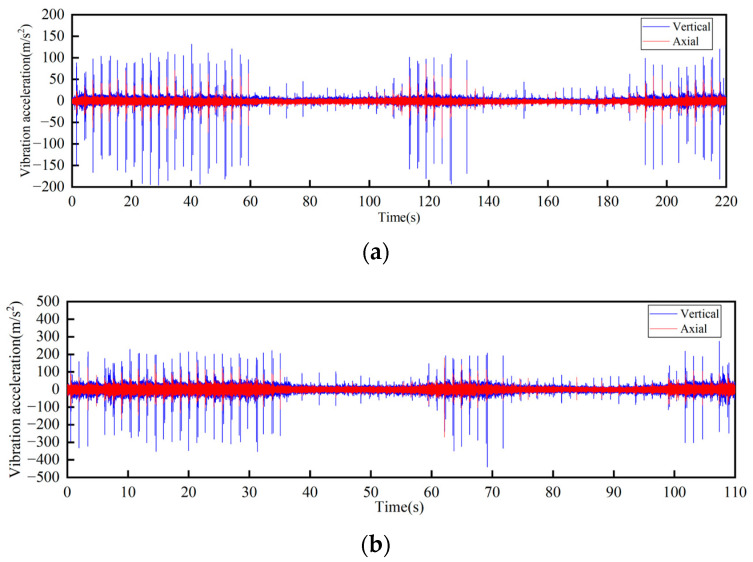
Time-domain curves of vibration acceleration at the weld steps of rail joint under different operating speeds: (**a**) Speed 1 m/s; (**b**) Speed 2 m/s.

**Figure 16:**
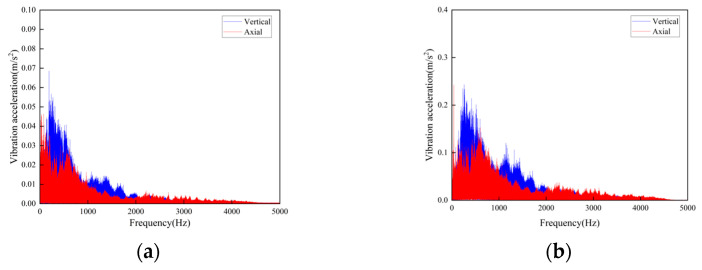
Frequency-domain curves of vibration acceleration at the weld steps of rail joint under different operating speeds: (**a**) Speed 1 m/s; (**b**) Speed 2 m/s.

**Figure 17:**
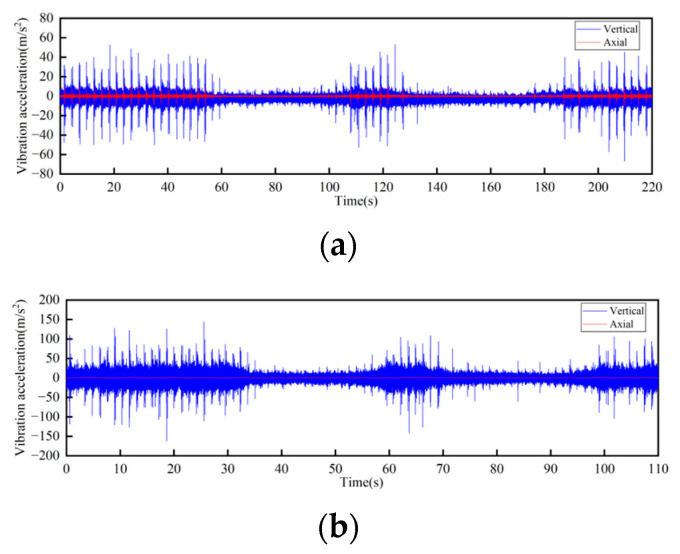
Time-domain curves of vibration acceleration at the flat weld of rail joint under different operating speeds: (**a**) Speed 1 m/s; (**b**) Speed 2 m/s.

**Figure 18:**
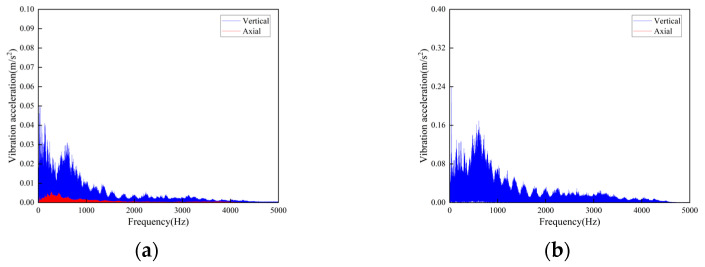
Frequency-domain curves of vibration acceleration at the flat weld of rail joint under different operating speeds: (**a**) Speed 1 m/s; (**b**) Speed 2 m/s.

**Figure 19:**
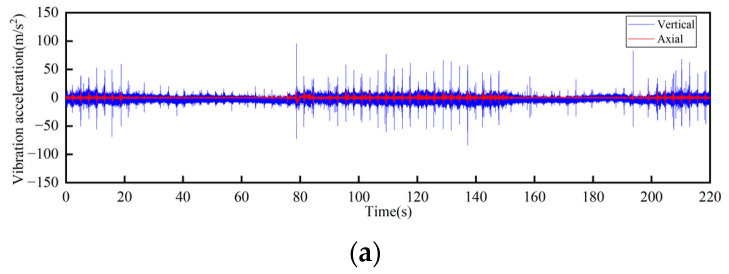

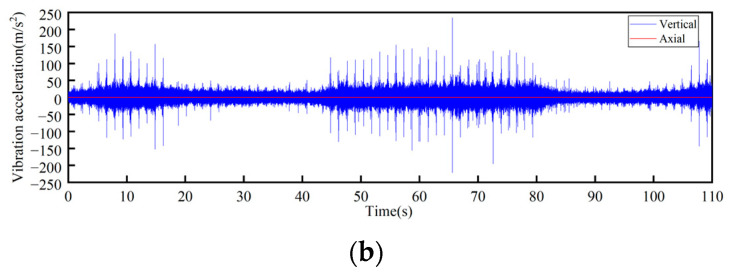
Time-domain curves of vibration acceleration at the relatively larger weld of rail joints under different operating speeds: (**a**) Speed 1 m/s; (**b**) Speed 2 m/s.

**Figure 20:**
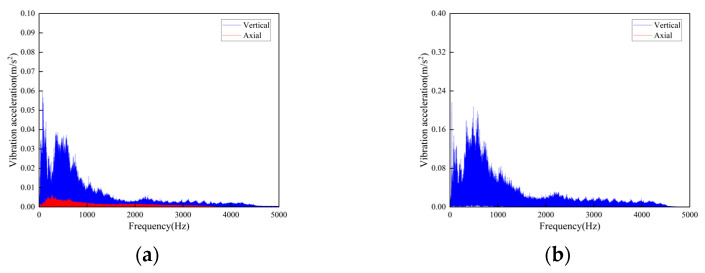
Frequency-domain curves of vibration acceleration at the relatively larger weld of rail joints under different operating speeds: (**a**) Speed 1 m/s; (**b**) Speed 2 m/s.

The track connections of the rail conveyor are welded. With the influence of service life and environment, cracks will occur in the welds. The measurements were conducted on the welds of three different cases, i.e., relatively flat welds, relatively large welds, and the uneven steps caused by weld fracture, and each place was installed two sensors in the axial and vertical directions, respectively. Two DH131E sensors were installed at the place of the weld fracture step, as seen in Figure 14 at ① and ②. For each case of the relatively flat and the relatively large welds, two sensors with different precisions, DH131E and AC133-1D, were respectively installed in the axial and vertical directions, as seen in Figure 14 at ③, ④ and ⑤, ⑥. The installation position information of each measuring point sensor is listed in Table 2, and the on-site measurement point layout at different joints of the track is shown in Figure 14. By performing Fourier transform on the measurement results, the vibration signals are analyzed in frequency domain and time domain, respectively. The measurement results are given in Figure 15, Figure 16, Figure 17, Figure 18, Figure 19 and Figure 20.

The following conclusions can be obtained by comparing the test data:

- For the three cases of the track joints, the vibration shock generated by the conveyor is distributed in the low-frequency band, mainly concentrated in the 0–1500 Hz area. The data output by the sensors will increase about three times when the conveyor speed is increased from 1 m/s to 2 m/s. The large fluctuations in the figure are the vibrations caused by the passing of the support carriage.
- Through the analysis of the vibration acceleration curve when the support carriage passes through, the vertical and axial vibration acceleration values of the track under the two operating speeds are all very large at the track weld step (Figure 14). This is because the track is in a completely disconnected state at the welded seam steps of the track, and the impact on both axial and vertical dynamic load is great when the carriage passes by.
- When the carriages passed through the places of the smooth and the relatively larger welding seams, the measured vibration acceleration curves showed similar trends. When the running speed was 1 m/s, the measured axial vibration acceleration of the track was about 10 times that of the vertical direction. As the operating speed increases, the vertical vibration acceleration decreases, which could be ignored when the operating speed is 2 m/s.

### 4.2. The Influence of Conveyor Track Parameters on Wheel-Track Noise

In this section, combined with the above analysis results, the influence of various track parameters including speed and fastener stiffness on the noise of the rail conveyor is discussed, which provides references for noise control of the system. The operating speed of the belt conveyor is relatively low compared to other wheel-rail mechanisms. When analyzing it in the frequency domain, it is found that the noise and vibration generated are mainly concentrated in the low-frequency area, so we chose the 0–2 kHz region for analysis.

1. The influence of conveyor running speed on the wheel and track noise

According to the wheel and rail parameters given above, the sound pressure levels of the rail conveyor at the operating speeds of 1 m/s, 1.5 m/s, and 2 m/s were predicted. The test results show that the noise levels at the head and the tail sections of the system are higher than that in the middle section but tend to be consistent with the increase of speed, so only the middle section was taken for study. The predicted noise levels of the middle section of the conveyor at different operating speeds are given as follows.

**Figure 21:**
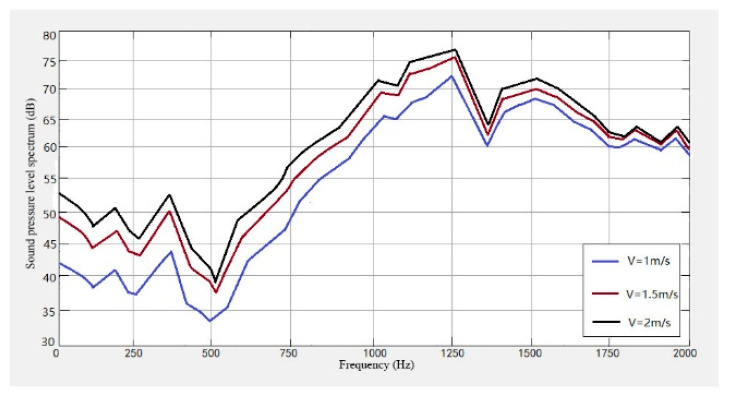
Wheel-track noise level spectrum at different speeds.

**Figure 22:**
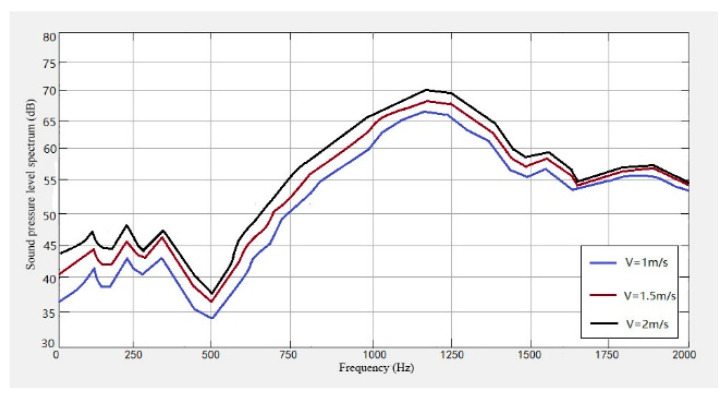
Wheel noise level spectrum at different speeds.

**Figure 23:**
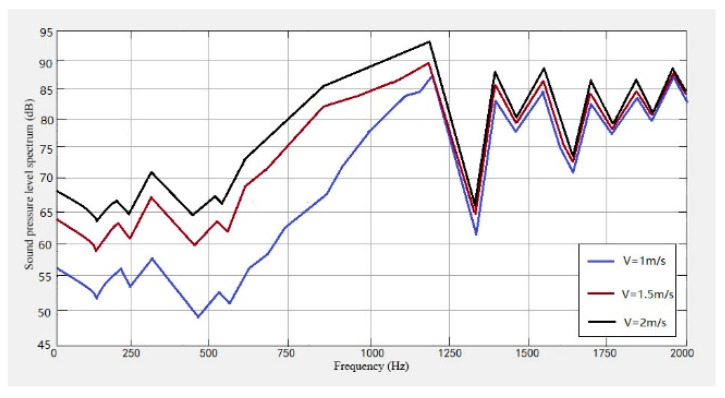
Track noise level spectrum at different speeds.

It can be seen from Figure 21, Figure 22 and Figure 23 that the conveyor wheel-rail noise, the carriage-wheel noise, and the track noise all increase with the increase of speed. When the speed increases from 1 m/s to 1.5 m/s, the noise level increases by about 7 dB, and when the speed increases from 1.5 m/s to 2 m/s, the noise level increases by about 4 dB on average.

**Figure 24:**
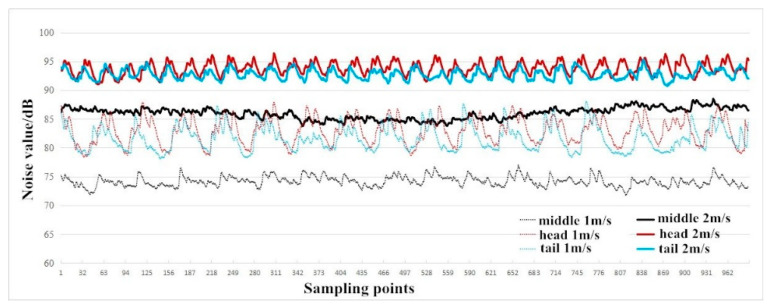
Noise values of head, tail, and middle sections under the speeds of 1 m and 1.2 m, respectively.

**Figure 25:**
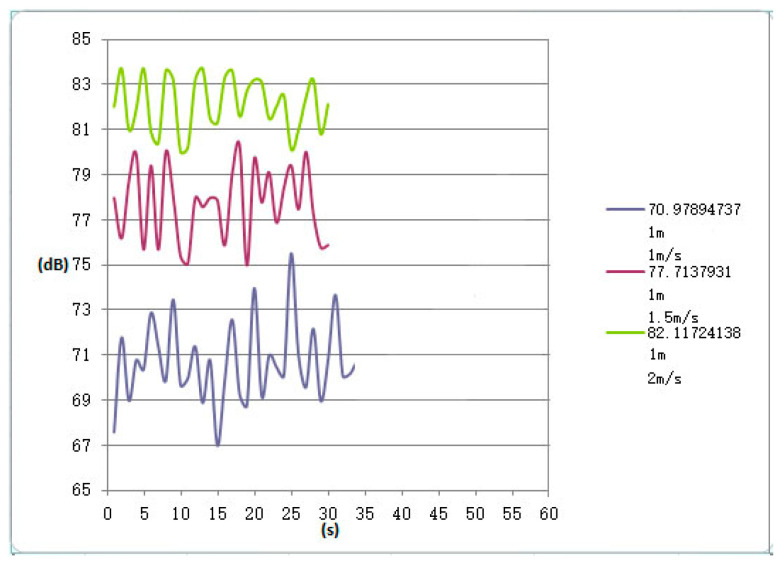
Variations of noise with time at the conveyor’s head under different operating speeds.

Figure 24 and Figure 25 show the measured noise curves on site. The on-site measurement environment and steps are as follows: The distance between the conveyor supporting carriages is 2 m, which can be operated cyclically on the track through the vertical turnaround wheels at the head and the tail of the system; the carriage-wheel material is cast iron; the parallel distance between the measuring point and the track is 1 m, the height of the measuring point is 1.2 m, and each test time is 30 s. The noise levels of the conveyor head, tail, and middle sections were then measured and analyzed, respectively, under several different operating speeds. Figure 24 shows the measured noises of the head, the tail, and the middle sections at operating speeds of 1 m and 1.2 m, respectively. It can be seen that the noise values at the head and the tail sections fluctuate more obviously. The peaks of the noise curves in Figure 24 are the noises measured when the supporting carriages were passing the measuring point, while the curve troughs the noises are measured when the previous carriage left and the next carriage had not reached the measuring point. Figure 25 shows the variations of the noises with time at the conveyor’s head part under different operating speeds. It can be seen that there are obvious vibration peaks when the supporting carriages pass the measuring point.

From Figure 21, Figure 22, Figure 23, Figure 24 and Figure 25, it can be seen that as the operating speed increases, the noise levels of wheels and tracks also increase. In the measured data, when the speed increases from 1 m/s to 2 m/s, the noise value increases by about 10 dB, which is consistent with the predicted value.

2. Influence of conveyor track fastener stiffness on wheel-rail noise

**Figure 26:**
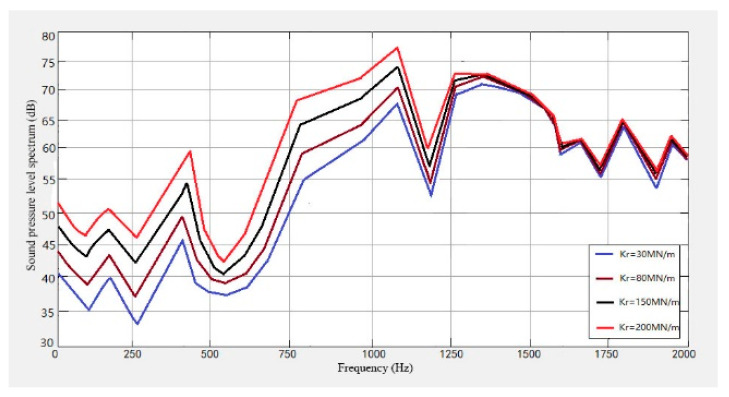
Wheel-track noise spectrum with different fastener stiffness.

**Figure 27:**
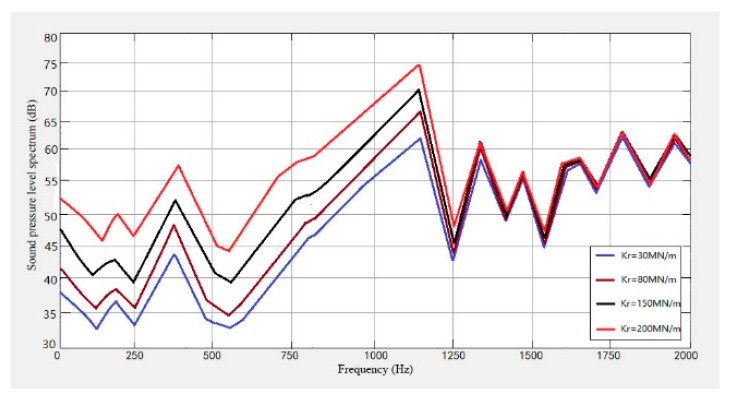
Wheel noise level spectrum with different fastener stiffness.

**Figure 28:**
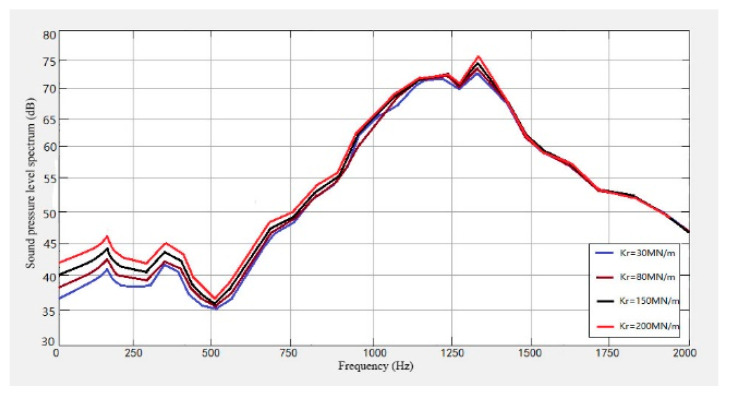
Track noise level spectrum with different fastener stiffness.

In order to study the influence of the fastener on the wheel and the track noise, four kinds of fasteners with stiffness of 30 M N/m, 80 M N/m, 150 M N/m, and 200 M N/m were selected, and the noise levels caused by them were predicted. The results obtained are shown in Figure 26, Figure 27 and Figure 28.

It can be seen from Figure 26, Figure 27 and Figure 28 that the noise of the system increases with the increase in the fastener stiffness under the stiffness of 30 MN/m, 80 MN/m, 150 MN/m and 200 MN/m. When the frequency is lower than 30 Hz, the stiffness has little effect on the noise. In the range of 30 Hz–1000 Hz, the noise sound level will increase with the increase in stiffness. When the frequency is greater than 1500 Hz, the track noise spectrum curve tends to be stable, and the stiffness has no effect on the noise.

## 5. Improvement of System Vibration and Noise

The dynamic interaction and vibration between tracks and wheels are the main noise sources of the rail conveyor. Therefore, controlling the conveyor wheel-track rolling noise is the key to reducing vibration and noise. The following measures can be taken to reduce the intensity of the vibration source, reduce acoustic radiation, or block the spread of noise.

1. Reduce the surface roughness of wheels and tracks

The wheel-track interaction is caused by the uneven surface of the wheels and tracks. Keeping the wheel-track surface smooth can effectively reduce the wheel-track interaction, thereby reducing wheel-track vibration and noise radiation. The wheel tread and track surface are polished to improve the surface finish. In addition, lubricating oil should be regularly added to the contact part of the wheel flange and tracks to effectively reduce the wear.

2. Laying seamless rails

Rail joints are an important cause of wheel-track impact noise. Due to the vertical and axial uneven problems at the rail joints of the prototype rail conveyor, the use of seamless rails to eliminate rail joints can effectively reduce the vibration and noise caused by wheel-rail impact.

3. Rail damper

**Figure 29:**
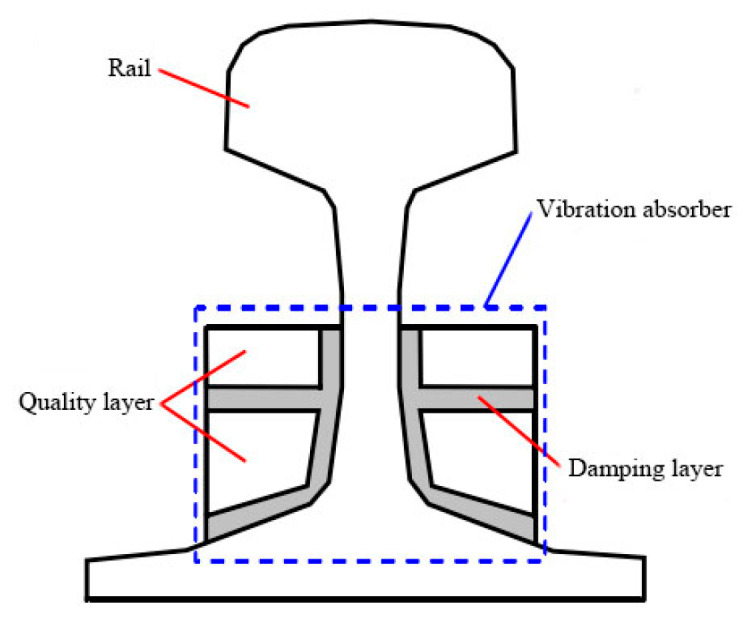
The sectional view of a rail damper.

The rail damper is composed of a mass block, a damping layer connecting the mass block and the rail [30], as shown in Figure 29. It has advantages of small quality, low cost, simple structure, and easy installation. Its working principle is mainly to form a mass-spring system through a thicker damping layer and a mass block to increase the attenuation rate of vibration along the rail, thereby effectively reducing noise. The rail damper can be directly pasted or fixed on both sides of the rail waist continuously or at intervals along the line with special clamps.

4. Set up sound barriers

A sound barrier made of unclosed dense materials can be used to block the sound waves between the noise source and the receivers, so that the sound waves are not easily transmitted, and the effect of reducing wheel and track noise is achieved. According to the actual situation, a layer of sound-absorbing material can be added to the side of sound barriers facing the sound source to further reduce noise. In practical applications, if the rail conveyor needs to pass through villages or very close to residential areas, sound barriers can be set up along both sides of the line to reduce the transmission rate of noise.

## 6. Conclusions

In this paper, a theoretical model of the rail conveyor prototype is established, and the field test is carried out. The test data are analyzed near the head and the frame track and the different welds of the track. Based on the analysis of wheel-rail vibration characteristics, the wheel-rail radiation noise is analyzed, and the sound pressure level of the conveyor at different operating speeds, track fastener stiffness, and different wheel-rail materials is predicted. Through experimental analysis, the following conclusions are drawn:

- At the same speed, the vibration of the truss connection is more severe than that of the reversing wheel. This is mainly due to the impact generated during the process of the supporting vehicle changing from contact with the upper track to contact with the lower track after turning, and the impact strength increases with the speed. At the same running speed, the vertical and horizontal vibration amplitudes are basically the same.
- At different welds of the track, the width and depth of the rail gap have a great influence on the vibration impact, which is mainly due to the impact of the uneven impedance at the track gap, and the greater the running speed, the more obvious the vibration impact. This phenomenon is especially pronounced at larger welds. At the relatively flat weld, the vertical vibration can be ignored with the increase in speed.
- In the process of comparing the actual test signals, the prediction model can analyze the running noise of the conveyor very well. At different wheel-rail speeds, the noise prediction results show that as the speed increases, the conveyor noise increases in turn, and the trend of increase gradually tends to be gentle.
- The noise level increases with the increase in the fastener stiffness in general. However, when the frequency increases to a certain degree, e.g., greater than 1400 Hz, the fastener stiffness seems to have little effect on the noise level.

In future work, it is also necessary to consider the influence of the different trolley spacing, different load operating conditions, the condition of the wheels of the supporting vehicle, and wheel-rail material on the vibration and noise of the system in the design process of the rail conveyor. The vibration of the inner and outer sides of the system turning section, the track under the head steering wheel under different trailer spacing, and different material conditions will be further considered.

## Figures and Tables

**Table 1:** Information of sensor installation on the track and truss near the system head.

**Serial Number**	**Name**	**Serial Number**	**Installation Location**	**Testing Direction**	**Running Speed**	**Sampling Frequency**
1	DH131E	①	Side of vertical turnaround wheel	Vertical	1 m/s, 2 m/s	10 kHz
2	DH131E	②	Side of vertical turnaround wheel	Axial	1 m/s, 2 m/s	10 kHz
3	DH131E	③	Side of vertical turnaround wheel	Horizontal	1 m/s, 2 m/s	10 kHz
4	DH131E	④	Upper track truss connection	Vertical	1 m/s, 2 m/s	10 kHz
5	DH131E	⑤	Upper track truss connection	Axial	1 m/s, 2 m/s	10 kHz
6	DH311E	⑥	Lower track truss connection	Axial	1 m/s, 2 m/s	10 kHz
7	DH311E	⑦	Lower track truss connection	Vertical	1 m/s, 2 m/s	10 kHz

**Table 2:** Sensor installation at different rail joints.

**Serial Number**	**Name**	**Serial Number**	**Installation Location**	**Testing Direction**	**Running Speed**	**Sampling Frequency**
1	DH131E	①	Weld step	Vertical	1 m/s, 2 m/s	10 kHz
2	DH131E	②	Weld step	Axial	1 m/s, 2 m/s	10 kHz
3	DH131E	③	Relatively flat	Axial	1 m/s, 2 m/s	10 kHz
4	DH131E	④	Larger weld	Axial	1 m/s, 2 m/s	10 kHz
5	AC133-1D	⑤	Relatively flat	Vertical	1 m/s, 2 m/s	10 kHz
6	AC133-1D	⑥	Larger weld	Vertical	1 m/s, 2 m/s	10 kHz

## Data Availability

Not applicable.
